# Depression, COVID-19 Anxiety, Subjective Well-being, and Academic Performance in University Students With COVID-19-Infected Relatives: A Network Analysis

**DOI:** 10.3389/fpsyg.2022.837606

**Published:** 2022-02-10

**Authors:** José Ventura-León, Tomás Caycho-Rodríguez, Karim Talledo-Sánchez, Kenia Casiano-Valdivieso

**Affiliations:** Department of Health Sciences, Universidad Privada del Norte (UPN), Lima, Peru

**Keywords:** depression, COVID-19 anxiety, subjective well-being, academic performance, university students, COVID-19-infected relatives, a network analysis

## Abstract

This study aimed to examine the relationship between anxiety, depression, subjective well-being, and academic performance in Peruvian university health science students with COVID-19-infected relatives. Eight hundred two university students aged 17–54 years (Mean 21.83; SD = 5.31); 658 females (82%) and 144 males (18%); who completed the Patient Health Questionnaire-2, Coronavirus Anxiety Scale, Subjective Well-being Scale (SWB), and Self-reporting of Academic Performance participated. A partial unregularized network was estimated using the ggmModSelect function. Expected influence (EI) values were calculated to identify the central nodes and a two-tailed permutation test for the difference between the two groups (COVID-19 infected and uninfected). The results reveal that a depression and well-being node (PHQ1-SWB3) presents the highest relationship. The most central nodes belonged to COVID-19 anxiety, and there are no global differences between the comparison networks; but at the local level, there are connections in the network of COVID-19-infected students that are not in the group that did not present this diagnosis. It is concluded that anxious–depressive symptomatology and its relationship with well-being and evaluation of academic performance should be considered in order to understand the impact that COVID-19 had on health sciences students.

## Introduction

The COVID-19 pandemic has affected physical and psychological health, causing high morbidity and mortality rates worldwide (Qiu et al., 2020). In view of this, quarantines and isolation have been useful measures to contain the impact of the infection (Brooks et al., 2020); however, it has also made people to experience greater psychological distress (Pfefferbaum and North, 2020), negative emotions, fear, and uncertainty (Mertens et al., 2020; Schimmenti et al., 2020). Studies on the consequences of the COVID-19 pandemic on mental health indicated negative effects in the general population (Salari et al., 2020; Xiong et al., 2020); specifically, in university students (Son et al., 2020; Li et al., 2021). In this regard, a recent systematic review and meta-analysis research, which included 27 studies and the participation of 706,415 university students, indicated a prevalence of depression of 39%, anxiety of 36%, stress of 6.39–21.65%, post-traumatic stress syndrome of 2.70–32.74%, fear of 12.52%, and panic of 20.41% in this population (Li et al., 2021).

The mental health status of university students during the pandemic has been affected by exposure to COVID-19; specifically, by information about the disease in social networks that associated with demographic variables, such as gender and years, the study has caused a great impact (Cao et al., 2020; Tang et al., 2020). Likewise, a sense of instability has been caused by the interruption of face-to-face classes and the lack of family support in many university students (Copeland et al., 2021). Other stressors in the university students during the pandemic include prolonged periods of quarantine, fear of infection, frustration, boredom, financial loss, and others (Li et al., 2021). Moreover, during the pandemic, there was a heightened fear of becoming infected with COVID-19 or having a relative infected with COVID-19 (Choi et al., 2020; Khademian et al., 2021). In fact, one study indicates that having a relative infected with COVID-19 significantly predicts increased anxiety and depression in university students (Wang et al., 2020; Copeland et al., 2021) and that this population is three times more likely to experience symptoms of depression compared to students without relatives with this condition (Wang et al., 2020). Similar findings in the general population demonstrate that exposure to a relative with COVID-19 symptoms increases the amount of stress, anxiety, and depression in the individual (Mazza et al., 2020; Zhao et al., 2020). As depression demonstrates interconnectedness with well-being (Bartoszek et al., 2020; Blanco et al., 2020; Ceri and Cicek, 2021), it is timely to study self-esteem and suicide (Fonseca-Pedrero et al., 2021).

During the pandemic, students are concerned about their well-being (Cuschieri and Calleja Agius, 2020), which is significantly related to their learning outcomes (Fawaz and Samaha, 2021). In this sense, the presence of symptoms of anxiety and depression negatively affects academic performance (Bolatov et al., 2021). On the other hand, increased concern about academic performance has also been identified as contributing to increased levels of anxiety and depression in students during the COVID-19 pandemic (Son et al., 2020). Furthermore, the academic performance of university students may be affected by remote teaching due to limitations in acquiring the necessary technology and/or having adequate digital connections (Rudenstine et al., 2021).

Recent scientific evidence indicates that the psychological responses to the COVID-19 pandemic are complex and involve numerous interrelated factors (Taylor et al., 2020). In this sense, network analysis is useful for the study of the interaction between variables, allowing to evaluate the degree to which variables belonging to the same construct are reciprocally associated and the way in which different constructs interact with each other (Di Blasi et al., 2021). Network analysis has been widely used in research on mental health problems and psychopathology for some years (Robinaugh et al., 2020). Specifically, it has become a suitable method for the analysis of clinical variables (Costantini et al., 2015). The application of this methodology to health sciences, specifically mental health, makes it possible to assess how certain behaviors or symptoms are associated with others (Levinson et al., 2017; Blanco et al., 2020; Di Blasi et al., 2021). In fact, there is some research using the network approach in university students finding that in this population, worry, trouble relaxing, and depressed mood are core symptoms (Bai et al., 2021) and when an abusive condition exists, networks report links to negative mood, sleep, and concentration problems (An et al., 2021); as well as a reduced ability to tolerate distress is associated with increased symptoms of depression (Lass et al., 2020). Despite this, there are few studies of well-being in the university students using this methodology.

Even though network analysis studies have been conducted during the pandemic of COVID-19 (Abdul Karim et al., 2021; Zavlis et al., 2021), clinical features of depression and anxiety, as well as experiences of well-being may vary depending on sample characteristics, culture, socioeconomic status or stressors (Bai et al., 2021). This implies that the results of network analysis in a given group are not easily applicable to other groups. Therefore, network structures of anxiety, depression, or well-being nodes must be analyzed within a specific population. As above, compared to general adult samples, univeristy students have a high likelihood of experiencing stressors that reflect the effects of the current COVID-19 pandemic, which may trigger increased anxiety and depression in the university context (Wang et al., 2020; Copeland et al., 2021). However, to date, network analysis has not been used to evaluate the interconnections between positive or educational variables in university students of health sciences in Peru, nor in any other Latin American country.

Therefore, this study aimed to estimate network structure of anxiety, depression, subjective well-being and academic performance nodes in Peruvian university students of health sciences with COVID-19-infected relatives. Specifically, we sought to: (a) identify the interconnectedness between nodes; (b) identify core nodes; and (c) compare two networks according to whether or not students were diagnosed with COVID-19.

## Materials and Methods

### Participants

There were 802 university students aged 17–54 years (Mean 21.83; SD = 5.31); 658 females (82%) and 144 males (18%). The sample size was determined *a priori* using a Monte Carlo simulation-based method (Constantin et al., 2021) which indicated 300 observations as minimally recommended; thus, the study far exceeded the estimated number. The selected sampling was non-probability *snowball* sampling because it started with a small number of initial contacts who met the research criteria (e.g., having COVID-19-infected relatives), they recommended other potential participants, and so forth (Parker et al., 2020). For further details of the participants, see Table 1.

**Table 1:** Social demographic variables.

Variables	*f*	%
Sex		
Female	658	82.00
Male	144	18.00
Range of age		
≤19 years	305	38.10
20–22 years	277	34.50
≥23 years	220	27.40
Cycle		
Start (1–4)	394	49.10
Intermediate (5–6)	289	36.10
Final (7–12)	119	14.80
Diagnosis COVID-19		
Yes	223	27.80
No	579	72.20
Careers		
Nursery	91	11.30
Nutrition	105	13.10
Obstetrics	100	12.50
Psychology	414	51.60
Physical Therapy and Rehabilitation	92	11.50

The ranges of ages are made through quartiles.

### Instruments

*Patient Health Questionnaire* (PHQ-2; Yu et al., 2011) composed of two items with a response scale ranging from 0 to 3 (0 = No days, 1 = Several days, 2 = More than half of the days, 3 = Almost every day). The patient health questionnaire-2 (PHQ-2) is unidimensional and measures two main symptoms of depression. Higher scores indicate greater severity. The psychometric properties of the PHQ-2 were reviewed for the present study. Reliability was estimated by the omega coefficient (*ω*) which indicated acceptable internal consistency (*ω* = .74) and internal structure by confirmatory factor analysis (CFA) which revealed excellent goodness of fit (RMSEA = .00; CFI = 1.00).

*Coronavirus Anxiety Scale* (CAS; Lee et al., 2020) composed of five items with a five-point Likert-type response scale (0 = Not at all, 1 = Rarely, less than a day or two, 2 = Several days, 3 = More than 7 days, 4 = Almost every day in the last 2 weeks). The coronavirus anxiety scale (CAS) is a unidimensional scale that measures anxiety response to COVID-19, with higher scores indicating greater symptom severity. It has evidence of validity and reliability in the Peruvian context (Caycho-Rodríguez et al., 2020). In spite of that, reliability was estimated for the sample under study (*ω* = .79) and the internal structure by CFA considering only the items that were included in the network (item: 1, 3, 5) which showed an excellent goodness of fit (RMSEA = .00; CFI = 1.00).

*Subjective Well-being Scale* (SWB; Su et al., 2016) composed of three items with seven-point Likert-type response alternatives ranging from Strongly Disagree (1) to Strongly Agree (7). Subjective Well-being scale (SWB) is a unidimensional scale that measures the person’s cognitive appraisal of his/her own life as satisfactory and a positive experience. It has good psychometric properties (*α* = .87). In spite of that, reliability was estimated for the sample under study reporting good reliability (*ω* = .90) and internal structure (RMSEA = .00; CFI = 1.00) of the items that were included in the network (items 2 and 3).

*Self-reporting of Academic Performance* (SAP) A single measure based on the proposal of Dominguez-Lara (2017), which allows for a quick exploration of the student’s perception of his/her academic performance. This single-question form has shown a moderate relationship with the student’s grade (*r* = .48).

### Procedures

Initially, a research project was elaborated and accepted by the authors’ university, which considered the ethical aspects of the Helsinki declaration (World Medical Association, 1964). Due to the pandemic, data collection was done through the Internet [Internet Mediated Research (IMR; Hoerger and Currell, 2011)]. Therefore, before answering the questionnaires, participants responded to an informed consent form explaining the purpose of the study, privacy and confidentiality of the information collected and data treatment. The collection began with the sharing of a *Google form* link to a group of students from a faculty of health who met the research criteria of having had a relative infected with COVID-19; then, they were instructed to share the link with classmates who also met this criterion and successively increasing the sample size. The collection was carried out between 05-09-2021 and 23-11-2021. The questionnaire took an average of 10 min to answer.

### Data Analysis

Statistical analyses were performed with the R programming language in its RStudio environment (RStudio Team, 2020). The psychological network approach was used (Epskamp and Fried, 2018). Previously, the presence of redundant nodes (symptoms) was examined by checking pairs of highly related nodes (*r* > .50) through the *networktools* package (Jones, 2021). This is because redundant nodes distort the estimated network and centrality indices, such as expected influence (Hallquist et al., 2021).

The network was estimated with the *qgraph* library (Epskamp et al., 2012) and performed through a non-regularized partial network, using the *ggmModSelect* function; since the study is aimed to examine central symptoms and a Spearman correlation matrix due to the ordinal nature of the variables (Isvoranu and Epskamp, 2021), understanding that partial correlations establish correlations among other symptoms after controlling for the relationship with other variables (Epskamp and Fried, 2018).

The interpretation of the network is done by considering that each node (circles) is connected to other nodes by edges (lines) whose edge thickness denotes the strength of relationship. A green shade of the edge denotes positive relationship and a red shade denotes negative (Epskamp et al., 2018). The network was organized considering the Fruchterman–Reingold algorithm (*spring* command) whose main characteristic is to place the strongest correlations in the center and the weakest ones in the periphery (Fruchterman and Reingold, 1991). In the case of comparisons, a *circle* organization was used to facilitate the comparison by placing the nodes in the same place for both groups.

Node centrality was performed using the expected influence index (EI) which is appropriate when the network contains positive and negative edges, because strength centrality may not predict accurately in such situations (Robinaugh et al., 2016). Other centrality measures, such as intermediation and closeness, were not estimated because they were not considered appropriate for psychological networks (Bringmann et al., 2019). In fact, a simulation study showed that both are strongly affected by sampling variation, making their use in psychology untenable (Hallquist et al., 2021).

The accuracy and stability of the edge weights are examined through the bootstrapping technique with the *bootnet* package. First, the accuracy consisted of repeatedly estimating a model with the sampled data and estimating a value for the edges. This allows the calculation of 95% bootstrap confidence intervals (CI) whose amplitude denotes the stability of the edges (Epskamp, 2020). Second, the stability coefficient (SC), indicating the maximum proportion of cases to be removed was calculated before at least 95% of 1,000 bootstrap correlations of the true and resampled centrality indices are correlated by more than .70; thus, the SC is suggested to be above .50 and not below .25 (Epskamp et al., 2018).

Finally, two sub-networks (students who were infected by COVID-19 and those who were not) were compared using the NCT library (Van Borkulo, 2015). The NCT uses a two-tailed permutation test in which the difference between the two groups is calculated across 100 replicates for each randomly regrouped individual. The null hypothesis is that both groups are equal at .05 level of significance.

## Results

### Previous Assumption

Previously, we examined the presence of redundant nodes and found that the items CAS2 and CAS4, SWB1 were reiterative in the network. Therefore, they were removed before the estimation of the network.

### Estimate and Accuracy of the Network

**Figure 1:**
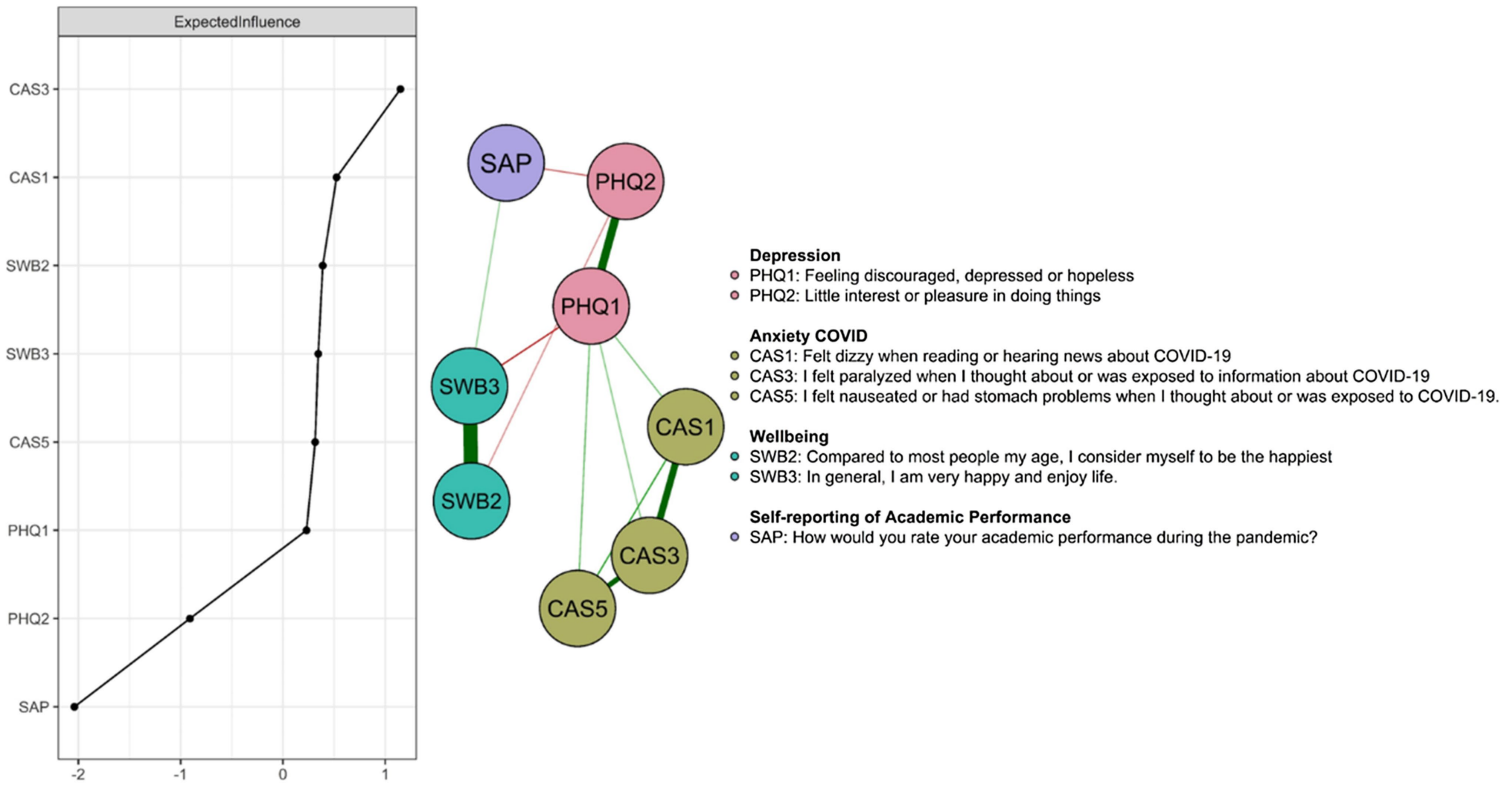
Depression, COVID-19 anxiety, well-being, and academic performance in university students with COVID-19-infected relatives. The EIs are shown in the left panel. The phrasing of the coronavirus anxiety scale items was shortened in order to enter them in the Figure; for complete information on the items, see Caycho-Rodríguez et al. (2020).

The *ggmModSelect* network for the data collected during the pandemic in university students are presented in Figure 1, and out of 11 possible nodes, only 8 were entered into the final model due to redundancy criteria. Symptoms from each theoretical community (PHQ, CAS, SWB, SAP) were clustered closer to each other. Average edge weights were found to be in the range −.18 to .76 and 12 of the 28 edges were non-zero. Intragroup edges presented positive and intergroup negative measures of association between mental disorders (CAS, PHQ) with well-being and performance. According to the network visualization each measure formed its own group. In particular, the strongest connection is presented by the PHQ1 node and SWB3 with a negative relationship.

### Centrality

The central nodes according to EI (see Figure 1) were CAS3 (“I felt paralyzed when I thought about or was exposed to information about COVID-19”), CAS1 (“I felt dizzy when I read or heard news about COVID-19”). Thus, the two COVID-19 anxiety nodes were among the most central; while the least central one is self-reporting of academic performance (SAP).

### Accuracy and Stability of the Network

**Figure 2:**
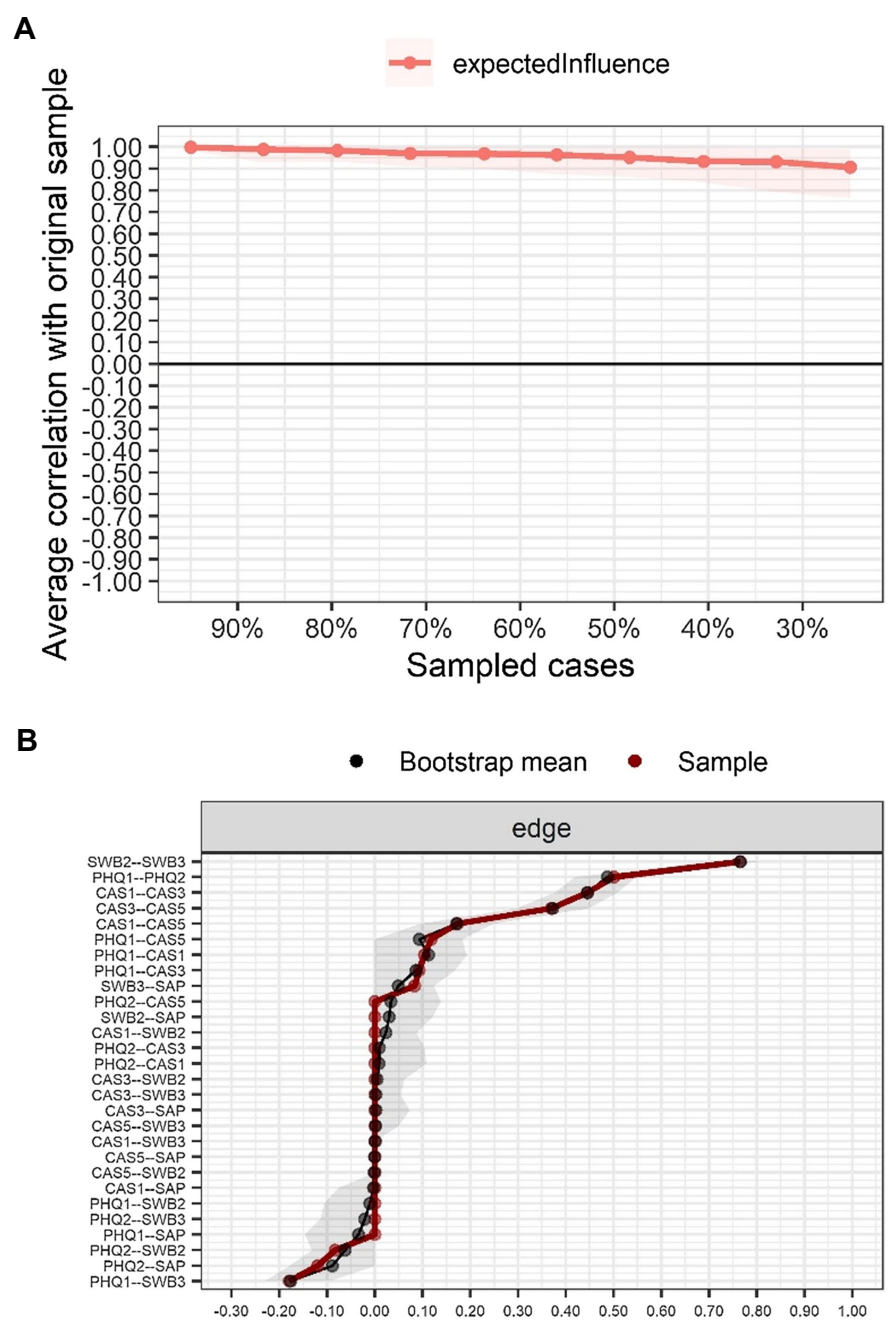
Stability and accuracy of the network. **(A)** Stability of the EI centrality index; **(B)** Accuracy through the non-parametric bootstrap confidence intervals (95%) of edges estimated for the network.

The stability statistic (Figure 2A) for the expected influence index confirms robustness (CS coefficient = .75); thus, robust network inferences can be made. Also, the 95% bootstrap CIs were not large, meaning that they did not vary significantly between resamples (Figure 2B).

### Comparison

**Figure 3:**
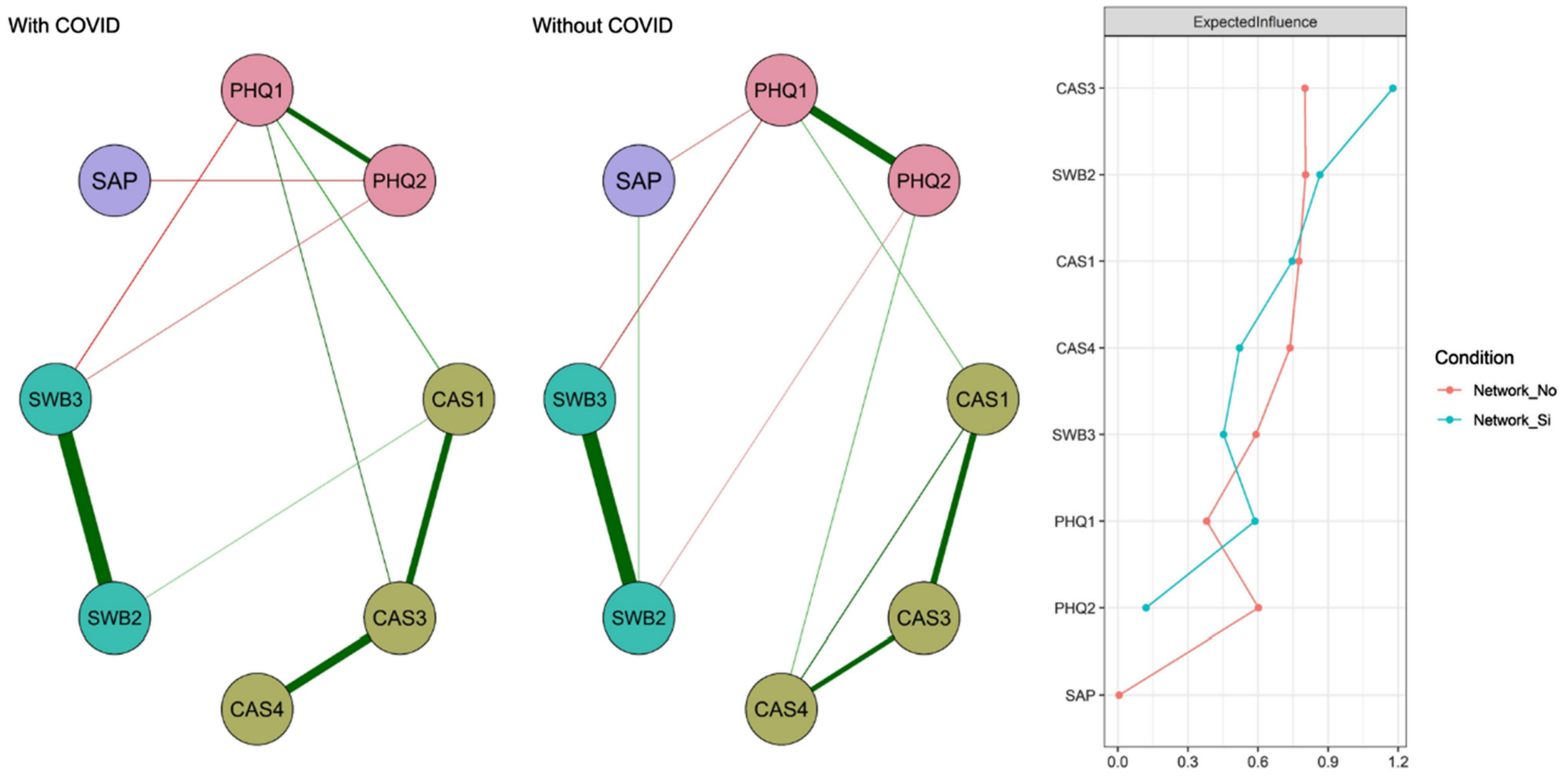
Networks according to the presence or absence of COVID-19 diagnosis in the student. Network_No indicates the centrality measure for the Without COVID-19 group and Network_Yes for the With COVID-19 group.

At a general level, the networks can be observed to be invariant (*M* = .21; *p* = .340) and that the level of connectivity is almost identical (*S* = .05; *p* = .760). Likewise, when the relationship between adjacent matrices is examined using Pearson’s correlation calculation they prove to be similar (*r* = .90). However, at the local level, the presence of connections between SAP-PHQ2, SWB2-CAS1, PHQ1-CAS3, and SWB3-PHQ2 nodes that are not present in the Without COVID-19 network is observed in the With COVID-19 network. Similarly, the relationship between SAP and PHQ2 is present in the Without COVID-19 network and does not appear in the other contrast network. Finally, the EI shows a greater difference in PHQ2 and PHQ1 (Figure 3).

## Discussion

This study is the first to examine the relationships between depression, COVID-19 anxiety, well-being, and academic performance in Peruvian university health science students with COVID-19-infected relatives; through network analysis. This approach allowed us to examine partial correlations between nodes, which consists of establishing relationships after controlling for the relationship between other variables (Epskamp and Fried, 2018). Thus, we were able to examine the interaction between the constructs (Di Blasi et al., 2021) related to mental health issues, such as wellness, COVID-19 anxiety, depression (Levinson et al., 2017; Blanco et al., 2020; Robinaugh et al., 2020), and self-evaluation of academic performance.

In this sense, the first objective was to identify the interconnection between the nodes, observing that nodes PHQ1 (“Feeling discouraged, depressed or hopeless”) and SWB3 (“In general, I am very happy and enjoy life”) presented the highest relationship. That is, depressed mood is related to the experience of enjoying life; these findings are related to previous studies (Bartoszek et al., 2020; Blanco et al., 2020; Ceri and Cicek, 2021). In fact, this is to be expected because a study of health professionals estimated that the prevalence of depression during the pandemic reached 23% (Bartoszek et al., 2020), This situation suggests that this clinical symptomatology was also present in university students of health sciences. These findings suggest the need for positive interventions in health science students, which, according to a previous study conducted from the network approach, proved to be effective in reducing depressive symptoms (Blanco et al., 2020). They also emphasize that university students faced emotional instability during the pandemic (Copeland et al., 2021), which together with prolonged quarantine, fear of infection, boredom, and economic losses (Li et al., 2021) may have increased psychological distress in health science students.

A second objective was to identify the central node in the network. Thus, the two central nodes belonged to the COVID-19 anxiety community, such as CAS3 (“I felt paralyzed when I thought about or was exposed to information about COVID-19”) and CAS1 (“I felt dizzy when I read or heard news about COVID-19”). These results indicate that anxiety about COVID-19 was found to be present as the most representative node in health science students. However, the study is not the first to demonstrate the importance of anxiety in university students during pandemic (Wang et al., 2020; Bai et al., 2021); but it is one of the first to examine from a network approach the relationships of this anxiety with depression, subjective well-being, and academic performance in health science students with relatives infected with COVID-19. In fact, this is important because previous studies in the general population of university students indicate that having a relative infected with COVID-19 significantly predicts increased anxiety and depression (Wang et al., 2020; Copeland et al., 2021) and increases three times more the risk of presenting depressive symptomatology (Wang et al., 2020).

A third objective was to compare the nodes according to whether the student presented COVID-19 or not. The findings indicate that globally the networks are invariant and the adjacent matrices are very similar. However, in the group of COVID-19-infected students, relationships appear between some nodes that are not present in the group of students who did not have COVID-19. First, SAP (“How would you rate your academic performance during the pandemic?”) with PHQ2 (“Little interest or pleasure in doing things”) this could reveal the impact of depressed mood on the evaluation of academic performance, which according to previous studies can be considered a central node (Bai et al., 2021), that together with some aggravating factors, for example, abuse in childhood may reveal the presence of sleep and concentration problems (An et al., 2021) negatively affecting academic performance and learning outcomes (Cuschieri and Calleja Agius, 2020; Fawaz and Samaha, 2021). Second, node SWB2 (“Compared to most people my age, I consider myself the happiest”) and CAS1 (“I felt dizzy when I read or heard news about COVID-19”); this would indicate that a good state of physical health is necessary for a satisfactory evaluation of well-being (Melnyk et al., 2020). Third, PHQ1 (“Feeling discouraged, depressed or hopeless”) with CAS3 (“I felt nauseous or had stomach problems when thinking about or being exposed to COVID-19”), suggesting that mood is highly correlated with being physically well; thus, people with illness demonstrate a greater predisposition toward depression (Melnyk et al., 2020). Fourth, SWB3 (“I am generally very happy and enjoy life”) with PHQ2 (“Little interest or pleasure in doing things”), which is expected to appear in the network of people diagnosed with COVID-19 and not in people without this diagnosis. In fact, some previous studies warned of these results (Bartoszek et al., 2020; Blanco et al., 2020; Ceri and Cicek, 2021). These findings are relevant because they point to the presence of relationships of abulia and well-being in the population of students having relatives with COVID-19. Finally, in the network of people who did not present COVID-19, there is a relationship between SAP (“How would you rate your academic performance during the pandemic?”) with PHQ1 (“Feeling discouraged, depressed or hopeless”), which is another symptom of depression. Here it is interesting to underline the existence of third variables in the performance evaluation, such as self-concept, self-esteem, and adverse experiences (Wong et al., 2019) that may have generated the absence of this interaction in the network of COVID-19-infected students.

The findings found present strong theoretical and practical implications. The fact that the core nodes of the network are COVID-19 anxiety provides support for models that emphasize the role of anxiety in the higher education setting (Wang et al., 2020; Bai et al., 2021). This is consistent with the substantial anxiety increase during the pandemic (Liu et al., 2021), which leads us to pay attention to anxiety in university faculties when blended learning is implemented in March 2022 in Peru. Secondly, it is interesting to examine the presence of greater interconnections of depressive symptoms in the group of students who were infected by COVID-19; apart from having had a relative infected by COVID-19, it warns of the need to provide protective factors for depression (Wang et al., 2020) because the conditions of personal adversity together with low tolerance to distress favor the presence of a negative mood, sleep, and concentration problems (An et al., 2021). In a practical way, it is expected that the results can be used to consider the anxious–depressive symptomatology in the prevention, diagnosis, and treatment plans of the psycho-pedagogical departments of the universities; which, as shown by this study, are related to the well-being and evaluation of academic performance. This is extremely important in health sciences students because once the containment measures are lifted and blended learning occurs in universities, they will be the most exposed to COVID-19 infection (i.e., professional practices and patient care support); ergo, an increase in anxiety toward COVID-19 infection (Bai et al., 2021). Finally, it is important to note that a COVID-19-infected family member affects the student’s well-being by increasing stress, anxiety, and depression (Mazza et al., 2020; Zhao et al., 2020). In fact, if the family is understood as a system, the affectation of one of its members can cause changes in the other members. Specifically, the care required by a sick family member demands high levels of stress and depression, and the unpreparedness of family members to provide such care causes emotional exhaustion (Sheth et al., 2021).

Despite the interesting findings, the study has certain limitations. First, the participants were selected with a non-probabilistic snowball design. This affects inferences and hinders the ability to generalize. However, in some cases, probabilistic designs are complicated, and non-probabilistic designs tend to be preferred (Fricker et al., 2019); In addition, collecting random data virtually is complex during the pandemic. Second, there is a wide difference between those diagnosed and those not diagnosed with COVID-19, due to the sample design; nevertheless, it would be advisable in future studies to work with equivalent groups. Third, there was a difference in the number of women and men, which is to be expected because the proportion of women is always greater than that of men in health sciences careers; however, in future studies that seek to compare by gender, it would be ideal to reduce the sample differences according to gender. Fourth, the test items were phrased to incorporate expression *during the pandemic…* generating a retrospective evaluation of the participants; this evaluation of past behavior may have caused a bias; but these modifications may still be relevant in cross-sectional studies (Blome and Augustin, 2015).

## Conclusion

In this paper, we examined the interaction between depression, COVID-19 anxiety, well-being, and academic performance in Peruvian university health science students with COVID-19-infected family members. The results indicate that the nodes of PHQ1 (“Feeling discouraged, depressed or hopeless”) and SWB3 (“In general, I am very happy and enjoy life”) presented the highest interconnectedness. The nodes with the highest centrality were also identified to be those pertaining to COVID-19 anxiety. In general terms, the networks behaved similarly in the comparisons; nevertheless, there are connections in the network of COVID-19-infected students that are not in the group that did not present this diagnosis. Finally, we encourage further examination of relationships on mental health variables in health sciences students to understand the impact of COVID-19 on this population.

## Data Availability Statement

The raw data supporting the conclusions of this article will be made available by the authors, without undue reservation.

## Ethics Statement

The studies involving human participants were reviewed and approved by Universidad Privada del Norte Ethics Committee. The patients/participants provided their written informed consent to participate in this study.

## Author Contributions

JV-L: conceptualization, investigation, methodology, supervision, formal analysis, and writing—original draft. TC-R: conceptualization, investigation, project administration, supervision, and writing—review and editing. KT-S: conceptualization, investigation, project administration, and supervision. KC-V: conceptualization, investigation, supervision, and writing—review and editing. All authors contributed to the article and approved the submitted version.

## Funding

This work was supported by Universidad Privada del Norte.

## Conflict of Interest

The authors declare that the research was conducted in the absence of any commercial or financial relationships that could be construed as a potential conflict of interest.

## Publisher’s Note

All claims expressed in this article are solely those of the authors and do not necessarily represent those of their affiliated organizations, or those of the publisher, the editors and the reviewers. Any product that may be evaluated in this article, or claim that may be made by its manufacturer, is not guaranteed or endorsed by the publisher.
